# Exploring the use of a youth specific screening tool in hematological cancer care: perspectives from young adult patients and healthcare professionals

**DOI:** 10.1007/s00520-026-10814-8

**Published:** 2026-05-28

**Authors:** Iben Husted Nielsen, Kristina Holmegaard Nørskov, Line Bentsen, Helle Pappot, Nina Toft

**Affiliations:** 1 Department of Hematology, Copenhagen University Hospital, Rigshospitalet, Copenhagen, Denmark; 2 Department of Haematology, Zealand University Hospital, Roskilde, Denmark; 3 Department of Oncology, Copenhagen University Hospital, Rigshospitalet, Copenhagen, Denmark; 4 Department of Clinical Medicine, University of Copenhagen, Copenhagen, Denmark

**Keywords:** Patient engagement, Communication tool, Young adults, Cancer

## Abstract

**Background:**

Adolescents and young adults (AYAs) with cancer experience substantial psychological distress and unmet supportive care needs. Routine screening with age-specific tools may improve the identification and addressing of concerns. The Adolescent and Young Adult Psycho-Oncology Screening Tool (AYA-POST) is a validated instrument, yet little is known about how AYAs and healthcare professionals (HCPs) experience its use in clinical practice.

**Objective:**

To explore how the AYA-POST supports AYAs in identifying, prioritizing, and communicating the health concerns most important to them and to examine HCP perspectives on patient engagement, communication, and decision-making.

**Methods:**

We conducted a qualitative study combining semi-structured individual interviews with AYAs (18–39 years) in a treatment trajectory for acute leukemia or malignant lymphoma and focus group interviews with physicians and nurses. Patients completed the AYA-POST prior to two consultations; interviews followed the second visit. Data were analyzed using reflexive thematic analysis.

**Results:**

Patients experienced that the AYA-POST facilitated self-reflection, preparation, and prioritization of concerns, enhancing communication with HCPs. The tool uncovered often-overlooked physical, psychological, and sensitive issues, including sexual health and emotional well-being, and promoted patient empowerment. HCPs reported that it expanded consultations beyond biomedical symptoms and encouraged patient-centered dialogue. Challenges included time constraints, variability in patient readiness, staff training needs, and practical issues with paper-based forms. Both groups highlighted the importance of flexible timing, multidisciplinary collaboration, and integration into routine workflows.

**Conclusions:**

The AYA-POST can strengthen patient-centered communication and empowerment in hematological AYA care. Successful implementation requires integration into clinical workflows, staff training, flexible timing, and systematic follow-up.

**Supplementary Information:**

The online version contains supplementary material available at 10.1007/s00520-026-10814-8.

## Introduction

Each year, approximately 1500 adolescents and young adults (AYAs) aged 18–39 are diagnosed with cancer in Denmark, with hematological malignancies accounting around 30% of all cases [1–3]. Although overall 5-year survival in this group approaches 85%, outcomes for acute leukemias remain approximately 60% [4]. Given their long life expectancy, AYAs experience a disproportionate long-term disease burden, including persistent psychological distress that often begins at diagnosis and continues years after [5–8].

AYAs are recognized as a distinct population with unique biological, clinical, and psychosocial needs, requiring holistic survivorship care [9–11]. Unmet supportive care needs are associated with higher distress, reduced quality of life (QoL), poorer mental health and physical outcomes, while early and sustained supportive care can mitigate long-term adverse effects [12–16]. However, evidence shows that substantial and diverse supportive care needs often persist and remain unmet years after diagnosis [17]. These findings underscore the critical importance of developing comprehensive survivorship care frameworks that systematically assess and address the persistent supportive care needs of AYA cancer survivors, with the aim of reducing long-term morbidity and enhancing overall QoL.

Effective, age-appropriate communication is central to patient-centered AYA cancer care, yet many patients lack opportunities to discuss their most salient concerns during consultations with their treating physicians [18–22]. Previous studies have identified common communication barriers between AYAs and healthcare professionals (HCPs). Key concerns such as fertility, sexual health, education, career development, family planning, physical and emotional well-being, financial challenges, and late effects are often inadequately addressed [19, 21, 23]. These concerns may contribute to underreporting of symptoms and concerns and can lead to reduced QoL, unmanaged side effects, treatment delays, and ultimately poorer survival outcomes [14, 24].

Systematic psychosocial screening has been recommended by the National Comprehensive Cancer Network (NCCN) in 2007, with the Adolescent and Young Adult Psycho-Oncology Screening Tool (AYA-POST) identified as a promising instrument [5, 25].

While quantitative studies support its use in clinical utility, limited qualitative evidence exists on how AYAs and HCPs experience its use in practice. This study therefore explores how AYA-POST supports AYAs in identifying and communicating priority concerns and examines HCPs’ perspectives on its impact on patient engagement, communication, and clinical decision-making.

## Methods

The study employed a qualitative design, combining individual semi-structured interviews with patients and focus group discussions with HCPs. The research was conducted at the Department of Hematology at Copenhagen University Hospital, Rigshospitalet, Denmark. This study was designed and reported in accordance with the COREQ (Consolidated Criteria for Reporting Qualitative Research) checklist [26].

### The adolescent and young adult psycho-oncology screening tool

The AYA-POST is a validated needs-assessment tool for AYAs aged 15–39 years affected by cancer [5]. It identifies psychosocial and supportive care needs across domains such as emotional wellbeing, practical and financial issues, relationships, fertility, education and employment, and physical symptoms. It comprises a checklist of concerns and a distress thermometer completed before consultations. It serves as both a screening tool and a communication aid to structure patient-HCP dialogue. For this study, a Danish version was used, adapted for cultural relevance.

### Participants and procedures

Eligible patients were aged 18–39 years diagnosed with acute leukemia or malignant lymphoma requiring intensive chemotherapeutic treatment or allogeneic hematopoietic stem cell transplantation (HSCT). Additional inclusion criteria were Danish language proficiency and written informed consent. Patients were eligible from one-month post-diagnosis or three months post HSCT, during active treatment (including maintenance therapy), or follow-up (4 to 12 weeks post treatment). Eligible HCPs included physicians and nurses with clinical responsibility for this patient population. Patient exclusion criteria included significant cognitive impairment or unstable medical conditions.

AYAs and HCPs were recruited by the primary investigators (KHN and IHN) in the outpatient clinic between December 2023 and November 2024. Patients were enrolled during routine visits and completed the AYA-POST prior to consultation, after which selected items were jointly reviewed with the physician. This procedure was repeated at two visits no more than six weeks apart. The AYA-POST consultations were integrated into existing clinical visits, with timing aligned to routine clinical care and no additional appointments scheduled. Following the second visit, patients participated in a semi-structured individual interview. The AYA participants could choose to be interviewed at home, at the research facility, or at the hospital. Focus group interviews with HCPs were conducted at the hospital after completion of all patient interviews.

### Data collection and analysis

Separate semi-structured interview guides were developed for patients and for HCPs based on a review of the existing literature, to identify the theoretical and analytic categories relevant for the research topics.

Interviews were transcribed verbatim and analyzed as the study progressed, enabling an iterative and reflexive analytical approach. Data were analyzed using Braun and Clarke [27] reflexive thematic analysis. The analysis moved recursively through six phases rather than linearly. First, in level one both researchers (KHN and IHN) who contributed equally across all six analytical levels read and re-read the transcripts to become familiar with the data and documented preliminary analytic observations. In level two, meaningful data features were systematically coded, and initial codes were developed. The researchers independently coded the transcripts and developed an initial coding framework, which was iteratively refined through discussion. Any discrepancies were resolved through consensus. Codes were then compared, discussed, and grouped into preliminary patterns of shared meaning in level three, and these candidate themes were in level four reviewed in relation to both the coded extracts and the full dataset. In level five, themes were then reviewed, defined, and named in preparation for the final synthesis. The final level six encompassed the integration of the analytic narrative with illustrative data extracts and situating the findings in relation to the study aim and relevant literature. Reflexivity was maintained throughout the analytic process through regular discussions between the researchers, critical reflection on their assumptions and positions, and iterative revisiting the data. The collaboration between the two researchers was used to broaden and deepen interpretation rather than to achieve coding consensus or inter-rater agreement.

### Ethical considerations

The study was conducted in accordance with the ethical principles outlined in the Declaration of Helsinki [28] and registered at the Data Protection Agency of the Capital Region of Denmark (file no.: p-2023–14883). We recognized the vulnerable nature of the patient population and took measures to minimize participation burden, ensuring that study activities did not interfere with treatment schedules or add undue stress.

## Results

In total, 23 patients were enrolled and completed individual interviews, and two focus group interviews with physicians (*n* = 6) and nurses (*n* = 3). Tables 1and 2 summarize participant characteristics.

**Table 1:** Demographic and cancer related clinical data of the AYA participants

Characteristics	Total (*n* = 23)
Gender, *n* (%)	
Female	10 (43.5)
Male	13 (56.5)
Median age, y (range)	25 (19–38)
Relationship (yes), *n* (%)	11 (48)
Children (yes), *n* (%)	3 (13)
Highest educational degree^a^, *n* (%)	
No high school degree	4 (17)
High school degree	8 (35)
2 year college	4 (17)
4 year college	4 (17)
Masters degree or higher	3 (13)
Occupation, *n* (%)	
Saleried employee	9 (39)
Unemloyed	2 (9)
Sickness benefits	2 (9)
Undergoing education	10 (43.5)
Current residence, *n* (%)	
Living with parents	7 (30)
Living alone in an apartment/house	5 (22)
Living with others in an apartment/house	9 (39)
College housing	2 (9)
Cancer type, *n* (%)	
Acute leukemia	17 (74)
Malignant lymphoma	6 (26)
Time since diagnosis, *n* (%)	
Under 1 years	22 (96)
1–2 years	1 (4)
Treatment regimen, *n* (%)	
Chemotherapy	17 (74)
HSCT^a^	6 (26)
Treatment status, *n* (%)	
In treatment	16 (70)
Follow-up^b^	7 (30)

^a^*HSCT* allogeneic hematopoietic stem cell transplantation

^b^4 to 12 weeks post treatment

**Table 2:** Health care professionals characteristics

Characteristics	Total (*n* = 9)
Gender, *n* (%)	
Female	8 (89)
Male	1 (11)
Clinical profession, *n* (%)	
Nurse	3 (33)
Physician	6 (67)
Experience working in hematology, *n* (%)	
5–10 years	4 (44)
≥ 10 years	5 (56)

Four main themes were identified: (1) fostering reflection and preparation, (2) bridging sensitive issues, (3) enabling empowerment through involvement, and (4) challenges with implementation.

### Fostering reflection and preparation

Patients emphasized how the AYA-POST communication tool promoted self-reflection. Completing the AYA-POST encouraged them to pause and consider their health and emotional well-being in ways they had not previously done. This reflective process helped uncover concerns that might have gone unnoticed, increasing awareness of both personal feelings and broader life circumstances.

*“It made it more clear for me… Or it made me think more about some things I perhaps hadn’t thought about myself.” (Patient 19)*

For HCPs, the range of patient concerns uncovered through the tool was unexpected. As one nurse noted:

*“I thought we were asking systematically, but we uncovered far more issues than I had expected. I was surprised by how much came to light.” (Nurse 2)*

Several patients reported that the tool enhanced communication with their HCPs and enabled them to prioritize and articulate concerns more effectively. Many emphasized that checking boxes helped clarify priorities, making it easier to communicate with the physician and optimize the use of limited consultation time. Some described experiencing memory problems and being able to document their concerns in advance helped alleviate anxiety about forgetting important issues, particularly during emotionally or cognitively challenging moments.

*“It has made things more manageable for me. While undergoing chemotherapy, I would often forget things very quickly. If I did not write them down, I would not have remembered all the issues I needed to discuss with the doctor.” (Patient 14)*

Reflecting on the tool beforehand helped many patients structure their thoughts and feel better prepared and empowered for dialogue with their HCPs.

*“Without the form, I might have arrived at the doctor’s appointment without having considered what we needed to discuss. It became clear that there were numerous physical and psychological issues we should address.” (Patient 17)*

Similarly, physicians acknowledged that using the tool facilitated more focused and meaningful consultations, fostering a more collaborative clinical encounter. It helped prioritize patient concerns and allowed flexibility in the consultation agenda. As one physician remarked, it enabled them to *“skip some of the usual topics, which we often cover every time, to make room for what the patient really wants to discuss.” (Physician 3).* While some physicians initially feared the tool might prolong consultations, many found that it streamlined discussions by guiding the conversation toward the most relevant issues. Several patients highlighted that continuity and the ability to track changes facilitated ongoing dialogue and promoted a shared understanding of progress or emerging concerns with HCPs. The follow-up component was also valued for providing insight into their health status over time.

*“I could see that things did not necessarily improve as treatment progressed. However, completing the same tool each time made certain issues more apparent. It became clear to me that my mental health had deteriorated over the course of the treatment.” (Patient 21)*

### Bridging sensitive issues

Many patients expressed that the tool facilitated the exploration of both physical and psychological aspects of their illness and treatment, enabling them to raise topics that might otherwise have been overlooked or unarticulated, especially sensitive issues such as sexual health, emotional well-being, mental health, and the impact of illness on personal relationships.

*“I think it’s easier for me with the tool. I had marked something about sex, but I don’t think I would have mentioned it if I hadn’t checked it on paper beforehand. Maybe some HCPs find it difficult to talk about these topics, but when it’s clearly listed, it ensures that the topic is discussed.” (Patient 8)*

The tool supported the expression of feelings and concerns, allowing for more comprehensive and nuanced discussions than were typically observed in routine consultations.

*“Normally, the medical consultation focuses on blood test results, upcoming appointments, and whether things are generally going reasonably well.” (Patient 7)*

Several patients emphasized that the tool had helped normalize conversations around topics often considered taboo within medical contexts, thereby fostering openness regarding intimate and personal concerns.

*“I needed a long time to gather the courage to mark reduced sexual desire, because it concerns intimate matters that I normally have not discussed with my doctor. This normalizes all the topics that are relevant and, I think, should be normal to talk about.” (Patient 14)*

Some patients noted, however, that personal comfort levels could still constrain disclosure, particularly in relation to highly sensitive topics. The HCPs similarly reported that the tool had expanded consultations beyond standard biomedical symptoms to encompass social, emotional, and practical concerns that were frequently overlooked in our standard consultations.

*“We focus a lot on the physical symptoms. Often, you may sense that there are also other issues, for example, social and psychological problems. But for my own part, it is not something I bring up very often.” (Physician 4)*

The HCPs observed that the tool had prompted patients to address feelings and experiences revealing a broader spectrum of needs than were typically captured in routine consultations.

*“I was perhaps a little bit surprised about what was difficult for them. The physical issues did not take up so much space. And that is actually what we talk about most of the time.” (Physician 3)*

### Enabling empowerment through involvement

The tool facilitated patient empowerment and active participation in healthcare discussions. Most patients reported that the tool enhanced their sense of involvement and agency, enabling them to take a more active role in their care. Many described feeling empowered to articulate concerns, set the agenda for consultations, and participate collaboratively in decision-making processes.

*“I think it legitimizes discussing issues beyond just the test results. After talking about the blood tests, that’s usually it, and then the doctor moves on. This tool allows for opening up a conversation about the issues that have been most pressing to me.” (Patient 8)*

Several patients emphasized that reviewing the tool together with their HCP fostered a collaborative dynamic, promoting shared understanding and joint goal setting. This approach contributed to actionable steps in their rehabilitation or treatment, enhancing their clarity regarding treatment plans, medication, and future health prospects.

*“We sat and went through it together, discussing what my problems were. I thought that was fantastic. We both talked it through, and we both reached an agreement on what should happen. Nothing could have been done better.” (Patient 1)*

At the same time, the HCPs expressed that when they handed out the tool, it created an expectation that the patients’ problems would subsequently be resolved and addressed. However, the majority of patients highlighted that being acknowledged in their concerns significantly contributed to overall satisfaction and engagement.

*“But just being able to put it into words and being acknowledged for what I feel is actually very nice, and then I can put it aside again.” (Patient 6)*

The tool appeared to shift consultations from a more traditional, one-sided interaction toward a participatory model, in which patients could actively influence the direction and content of discussions.

*“It was I who decided when we had finished discussing the item I had marked. I moved on to the next question only when I was ready. In this way, I determined the pace and conclusion of the conversation.” (Patient 6)*

HCPs similarly reported that the tool empowered AYAs by enabling them to prioritize topics that mattered most to them. They observed a marked increase in patient engagement, with patients appearing more focused during consultations and more confident in articulating their needs.

### Challenges with implementation

All HCPs agreed that for the tool to succeed, it should be systematically integrated into clinical routines and workflows rather than treated as an optional or extra task.

*“It has to be something we actually prioritize to do, not just something we dismiss by saying: I don’t have time for that.” (Nurse, 2)*

The practical implementation of the tool revealed challenges that affected its consistent and effective use. Some physicians identified barriers such as time constraints during consultations, which made it difficult to fully engage with the concerns raised by the patients. They also expressed concern that in-depth discussions on sensitive topics could prolong consultations beyond what was feasible within standard scheduling.

*“I felt constrained when a patient marked nearly all emotional items. Within a 20-minute consultation also covering physical issues and treatment, I was unsure how to address them.” (Physician 1)*

Some HCPs emphasized the need for training, particularly to address topics such as sexuality, emotional distress, or existential concerns, and expressed discomfort or uncertainty in managing these issues.

*“I didn’t feel prepared to handle all these issues, other than being able to listen a little.” (Physician 2)*

Collaboration between physicians and nurses was seen as essential to ensure that the tool functioned as a shared resource. Others emphasized the need for clear options and resources to offer patients, suggesting the development of a shared catalog of support services.

Both patients and HCPs emphasized that the tool’s relevance varied across the treatment trajectory. One patient described it as *“a snapshot” (Patient 4)*, with usefulness increasing as new challenges arose, and suggested engagement should depend on readiness. HCPs noted effectiveness also depended on personality, emotional readiness, and trust, with some recommending introduction after a few treatment sessions rather than at the initial consultation.

*“They typically need to complete two or three treatment sessions before issues emerge… the tool becomes meaningful only partway through treatment.” (Physician 1)*

The HCPs noted that young patients often forgot the paper form and suggested a digital version with automated reminders to improve completion, integrate into workflows, reduce administrative burden, and enhance coordination.

## Discussion

This study demonstrates that the AYA-POST communication tool supports reflection, facilitates the articulation of sensitive concerns, and enhances patient involvement in clinical practice. These findings underscore the potential of the AYA-POST to strengthen patient-centered communication while illustrating the need for systematic implementation strategies.

Both patients and HCPs observed a shift from traditional, clinician-driven conversations toward a more patient-centered approach in the study. This aligns with research demonstrating that structured approaches to addressing informational and psychosocial needs enhance empowerment, coping, and perceived involvement among AYAs with cancer [29–32]. Health-related empowerment defined as the ability to manage one’s own health and care decisions is a core component of person-centered care and is closely associated with self-advocacy, engagement, and improved clinical outcomes, supporting emotional well-being and confidence in communication with HCPs [33]. Consistent with our findings, patients described feeling more capable of articulating concerns, identifying priorities, and engaging collaboratively in treatment planning. Furthermore, given that many young adults continue to face significant challenges, ongoing support and timely information throughout the care trajectory is particularly pertinent [30].

Aligned with our findings, previous studies highlight the need for HCPs to acknowledge the variability in AYAs’ communication preferences, which may change according to factors such as emotional state, physical condition, and the timing of clinical discussions [18]. Thus, adopting flexible, patient-centered communication strategies responsive to individual needs, rather than uniform approaches, is important. However, our results also indicate that many HCPs feel insufficiently prepared to engage in conversations involving sensitive or taboo topics. This is supported by a recent scoping review showing that inadequate training leads to avoidance of sensitive issues and highlights the need for additional communication training [21]. Although several communication frameworks aim to prioritize AYAs’ concerns and promote person-centered care, structured and systematically implemented training programs for HCPs remain limited. Addressing this gap is essential to support effective communication and meet AYAs’ diverse informational and emotional needs in clinical practice [21].

Our findings suggest that the tool helped the patients to slow down, reflect, and prepare before the consultation. This was especially valuable in the context of treatment-related fatigue, cognitive difficulties, and emotional distress, where the tool made it easier for the patients to remember what mattered most on the day, to prioritize across competing concerns, and to enter the consultation with a clearer sense of their own agenda. In this respect, our results converge with work on the generic PCI in head and neck cancer and other adult cancer populations, where PCI prompt lists have been shown to uncover a broader spectrum of concerns than clinicians routinely elicit, to structure consultations, and to legitimize discussion of taboo or “non-medical” topics [34, 35]. As others previously have described, several patients reported limited confidence in initiating difficult conversations themselves, especially about sexuality, intimate relationships, fertility, body image, or psychological distress [36, 37].

Employing a structured conversation tool represents an effective strategy for normalizing topics that are frequently regarded as taboo, most notably sexuality, in clinical encounters with AYA patients. Empirical evidence supports that, in the absence of structured approaches, sexual health is frequently under-addressed and continues to be treated as a taboo subject in clinical practice [38–40]. Some clinicians remain uncertain or hesitant when addressing sensitive topics whether related to sexuality, mental health, identity development, or relationship concerns [41]. In a multicenter cohort study (*n* = 242) among HCP treating AYAs, only a minority regularly addressed sexuality and gender identity despite acknowledging their importance, citing discomfort, lack of awareness, and fear of inducing shame [42]. Similarly, AYAs report substantial barriers to discussing sensitive issues in clinical settings. In a recent qualitative study both patients (18–22 years) and practitioners identified embarrassment, assumptions, and uncertainty as barriers to discussing sexual and relational health [40]. Importantly, the participants found that structured communication-support tools made these conversations feel more acceptable and less taboo [40].

To support effective implementation, targeted communication and capacity-building are essential. A scoping review of nurse-patient discussions about sensitive issues identified short, focused training programs including role-play, communication frameworks, and structured assessment tools as effective strategies for increasing clinician confidence [43]. Organizational and leadership support is equally important. Integrating discussions of sensitive topics into routine care requires explicit prioritization, sufficient time allocation, and a clinical culture that regards psychosocial and sexual health concerns as central to holistic cancer care rather than optional extras [44]. Digital integration may further enhance feasibility [45]; several HCPs in our study suggested that a digital version with automated reminders would reduce administrative burden, improve completion rates, and facilitate workflow integration.

The qualitative design allowed for an in-depth exploration of experiences from multiple perspectives and the use of methodological triangulation strengthened the credibility of the findings. Individual interviews facilitated rich, personal accounts, while focus groups encouraged professional reflection and collective sense-making, generating complementary insights. However, some limitations should be acknowledged. Participants were recruited from a single institution and within a Danish healthcare context, which may influence the applicability of findings to other cultural or organizational environments. Patients who were more medically stable and able to participate may be overrepresented, potentially excluding the perspectives of those experiencing greater illness burden. In addition, the AYA-POST was not validated in a Danish population prior to this study, which might influence accuracy. Moreover, given the broad age range in the participants, future qualitative studies could further explore how age and developmental stage may shape experiences and preferences within AYA care, as well as consider the timing along the cancer trajectory. Despite these limitations, the study design was appropriate for the exploratory aims and provides a robust foundation for understanding the use and perceived value of the AYA-POST in clinical practice.

## Conclusion

The AYA-POST communication tool enhances patient-centered care by fostering reflection, facilitating discussion of sensitive issues, and supporting patient empowerment in hematology consultations. The AYA-POST is well suited to the developmental position of AYAs with hematological cancer with patients reporting greater clarity, agency, and engagement, while HCPs noted more focused and meaningful interactions. Overall, the AYA-POST shows promise in promoting participatory care for adolescents and young adults, but its effectiveness relies on organizational support and structured implementation.

## Supplementary Information

Below is the link to the electronic supplementary material.

ESM 1 Supplementary Material 1 (DOCX 18.4 KB)

ESM 2 Supplementary Material 2 (DOCX 18.0 KB)

ESM 3 Supplementary Material 3 (DOCX 18.2 KB)

## Data Availability

Due to the sensitive and identifiable nature of the qualitative interview data, and in accordance with participant consent and ethical approval conditions, the datasets generated and analysed during this study are not publicly available.

## Declarations

### Clinical trial registration number

Not relevant.
