# Supplementary material for: Exploring the use of a youth specific screening tool in hematological cancer care: perspectives from young adult patients and healthcare professionals
Source: Support Care Cancer. 2026 May 28;34(6):589. doi: 10.1007/s00520-026-10814-8 (PMC13219061; doi:10.1007/s00520-026-10814-8)
Supplement: Supplementary file 1 — Supplementary Material 1 (DOCX 18.4 KB) [file 520_2026_10814_MOESM1_ESM.docx]

**Interview Guide AYA-POST – for nurses**

|  | Interview questions | Additional questions |
| --- | --- | --- |
| **Expectations and practical use** | How was it to inform patients about the questionnaire and hand it out to them?  What expectations did you have regarding the use of the questionnaire beforehand?  What are your experiences with the design of the questionnaire in terms of usability?  How did you use the questionnaire in practice?  Were you present during the consultation?  Did you follow up on the patient’s responses before or after the consultation with the doctor?  Did you find it necessary to prioritize among the patient’s responses? | How did you experience the patients’ responses to it?  Looking back, how have these expectations changed?  For example, do you find it clear and manageable as a tool?  For example, as part of the preparation process  If so, how did that work?  If so, how did that work?  Were there concerns or needs that, in your experience, were not addressed during the consultation? |
| **The significance of the questionnaire as part of the consultation** | For those of you who participated in the consultation: How did you experience the consultations being different when the questionnaire was used?  For those of you who were not present during the consultation: Did you experience that anything was different?  What challenges or barriers have you experienced in relation to the questionnaire?  What benefits have you experienced?  How has using the questionnaire influenced your relationship with your patients?  To what extent did you feel you had the competencies needed to address the issues the patient had indicated in the questionnaire?  To what extent did you have to follow up on, or initiate, actions that the doctor had referred the patient to? | Did it affect the duration of the consultation?  Were there topics that emerged which you would not normally have discussed with your patients?  For you as nurses?  For the patient?  Could you give examples?  For example, referral to a psychologist |
| **Significance for the patient** | Do you have the impression that patients subsequently used the advice or guidance you were able to provide, and did it make a difference?  Did it make a difference where the patient was in their treatment trajectory in terms of how the consultation unfolded and how the questionnaire was used? |  |
| **Looking ahead** | How do you see the questionnaire being used most effectively in the future?  Are there any practical circumstances that need to be considered?  Are there any considerations regarding the implementation process? | For example, frequency, timing, etc. |

Øverst på formularen

Nederst på formularen
