# Supplementary material for: Exploring the use of a youth specific screening tool in hematological cancer care: perspectives from young adult patients and healthcare professionals
Source: Support Care Cancer. 2026 May 28;34(6):589. doi: 10.1007/s00520-026-10814-8 (PMC13219061; doi:10.1007/s00520-026-10814-8)
Supplement: Supplementary file 2 — Supplementary Material 2 (DOCX 18.0 KB) [file 520_2026_10814_MOESM2_ESM.docx]

**Interview Guide AYA-POST – for patients**

|  | Interview questions | Additional questions |
| --- | --- | --- |
| **The questionnaire as preparation for the consultation** | How was it for you to complete the questionnaire about your concerns and burdens?  Which topics were relevant to you?  Were there any topics that you did not know, or did not think, you could discuss with your doctor? If so, please elaborate.  How did you experience the questionnaire as preparation for the consultation with the doctor and nurse? | What did you see as advantages or disadvantages?  Did completing the questionnaire lead to more concerns or fewer concerns? Please elaborate.  Did you find it meaningful to complete the questionnaire?  How did you experience answering the questions?  Did you find the questionnaire clear and easy to complete?  Were there any topics that seemed unnecessary? Please elaborate. |
| **The questionnaire as part of the consultation** | How did you experience the way the questionnaire was used by the doctor and nurse during the consultation?  Did you feel that the doctor had prepared for the consultation based on your responses?  Could you elaborate on whether your concerns were addressed during the consultation?  Did you feel involved in the consultation?  Did you feel that you were given advice on how to manage any concerns you might have had?  How, and to what extent, were your expectations of the consultation with the doctor and nurse met? | Which concerns were discussed?  Were any of your concerns or needs not addressed during the consultation? If so, please elaborate.  Did you experience consultations that included the questionnaire differently from other consultations with the doctor? If so, please elaborate.  How did you experience your own influence on the topics discussed during the consultation  Please elaborate and give examples. |
| **After the consultation and between consultations** | Have you used the advice or guidance you received during the consultation?  Has the questionnaire led you to focus more on your needs and concerns, and on how to manage them?  Is there anything you would like to have been different about the consultations? |  |
| **Closing** | Is there anything we have not discussed that you consider relevant and would like to add?  Thank you for your participation |  |

Øverst på formularen

Nederst på formularen
